# Understanding Acupuncture Based on ZHENG Classification from System Perspective

**DOI:** 10.1155/2013/956967

**Published:** 2013-11-21

**Authors:** Junwei Fang, Ningning Zheng, Yang Wang, Huijuan Cao, Shujun Sun, Jianye Dai, Qianhua Li, Yongyu Zhang

**Affiliations:** Center for Traditional Chinese Medicine and Systems Biology, Shanghai University of Traditional Chinese Medicine, Shanghai 201203, China

## Abstract

Acupuncture is an efficient therapy method originated in ancient China, the study of which based on ZHENG classification is a systematic research on understanding its complexity. The system perspective is contributed to understand the essence of phenomena, and, as the coming of the system biology era, broader technology platforms such as omics technologies were established for the objective study of traditional chinese medicine (TCM). Omics technologies could dynamically determine molecular components of various levels, which could achieve a systematic understanding of acupuncture by finding out the relationships of various response parts. After reviewing the literature of acupuncture studied by omics approaches, the following points were found. Firstly, with the help of omics approaches, acupuncture was found to be able to treat diseases by regulating the neuroendocrine immune (NEI) network and the change of which could reflect the global effect of acupuncture. Secondly, the global effect of acupuncture could reflect ZHENG information at certain structure and function levels, which might reveal the mechanism of Meridian and Acupoint Specificity. Furthermore, based on comprehensive ZHENG classification, omics researches could help us understand the action characteristics of acupoints and the molecular mechanisms of their synergistic effect.

## 1. Introduction

Acupuncture is an efficient therapy method that originated in ancient China. It uses thin metal needles to pierce through skin into acupoints to regulate the flow of Qi around the whole body, and its effect is validated primarily by the evidence of its beneficial practice while the mechanism awaits understanding. Acupuncture effect is complicated, which is determined by the complexity of human body and disease and is manifested by the properties that acupuncture acting factors possess multielement, multilevel, and nonlinearity [1]. But one of the problems in acupuncture research is how to study the action mechanism of acupuncture and evaluate its efficacy at molecular level based on scientific methods under the guidance of TCM theory. Reductionism study is unable to embody the integration and complexity of acupuncture effect; therefore, it is limited to reveal the rule of acupuncture effect without the guidance of systems theory.

As the most important part of TCM, acupuncture develops its therapeutic effect by stimulating acupoints, which could form a complex regulating network system through the flowing and changing of energy and information in meridians and collaterals [2]. The study of acupuncture should be based on the understanding of the complexity in TCM. ZHENG classification (also referred to as syndrome differentiation) is the essence of TCM which attaches importance to various factors, at the same time it emphasizes on the interaction and relation in integration, which is confronted with nonlinear phenomenon [3, 4]. The acupuncture research based on ZHENG classification is the systematic research which is under the guidance of TCM theory and could bring the complexity of acupuncture effect to light, so the acupuncture research on the foundation of ZHENG classification should be taken as the breakthrough point to reveal the acupuncture mechanism and its efficacy.

ZHENG is the specific pattern to identify disease and is the essence of ZHENG classification and treatment (Bianzheng lunzhi) in TCM [5], Sun et al. [6] use metabonomic methods to differentiate ZHENG types and evaluate the therapeutic efficiency of Fuzhenghuayu tablet in hepatitis-B-caused cirrhosis. The efficiency of FZHY treatment based on ZHENG differentiation indicated that accurately ZHENG differentiating could guide the appropriate TCM treatment in HBC. ZHENG is a relational model of dysfunctions in whole which is produced by logical reasoning based on practical experiences [7], and the nature of ZHENG may be the substances imbalance of multisystem and multilevel in spatiotemporal distribution and relation combination [8]. The idea of system biology is comprehensive, integrated, and global, which is in correspondence with the holistic and systemic approaches of TCM. As the coming of the system biology era, the system perspective provides us an important approach to understand the essence of phenomenon [9], and broader technology platforms were established for the objective study of TCM. Omics technologies could dynamically determine molecular components of various levels and creates the feasible conditions to explore the material basis of ZHENG. Lu et al. [10, 11] used genechip analytical techniques to distinguish between cold syndrome and heat syndrome of female rheumatoid arthritis (RA) patients; the results showed that the genes referred with cold or heat ZHENG were different from those genes with RA and further suggested that ZHENG in TCM has solid foundation in gene profile. Liu et al. [12] used proteomics technology to get the preliminary evidence in functional protein level that the transfer of phlegm and blood stasis syndrome is mainly from phlegm syndrome to blood stasis syndrome and ultimately formed phlegm accumulating with stagnation syndrome. In omics researches of ZHENG, system models have been used to analyze the interrelation between various factors in the whole. Xu et al. [13] explored a strategy of classifying five TCM syndromes in diabetes based on plasma fatty acid metabolic profiles, lipid metabolism indicators, and chemometrics methods. Compared with orthogonal signal correction-partial least squares (OSC-PLS) method, better clustering results were demonstrated with the application of the uncorrelated linear discriminant analysis (ULDA), a new method which was used to analyze the various factors in a joint way. By finding out the relationship between various response parts, omics researches can show us a systematic understanding of the microenvironment, based on the results of genomics research and literature data mining. Li et al. [14] successfully established a heat and cold ZHENG model.

Omics provides integral, systemic, and dynamic technology platforms for the study of TCM, the study by which has effectively revealed the essence and connotation of TCM phenomena such as ZHENG at molecular level. In the study of acupuncture, omics researches are helpful to understand the entire effect of acupuncture; moreover, based on ZHENG classification, holistic study can investigate the action characteristics of acupoints and the molecular mechanism of their synergistic effect under the guidance of TCM theory. So the present paper reviewed the omics researches of acupuncture. We hope that the review will help to understand the entire effect of acupuncture and the special effects of meridians and acupoints with combining the theory of TCM and help promote acupuncture to be more properly used clinically.

## 2. Omics Researches of Acupuncture Based on the Cognition of Diseases

Acupuncture stimulation could cause synchronous changes of body systems [1]. If we ignore the relation of the body systems and study the action mechanisms of acupuncture separately, we may offend against the view of holism and overlook the effective links. While the development of omics and bioinformation analysis technologies provide a global, systematical, and dynamic technology platform for TCM research, by dynamically detecting molecular components of various levels, omics researches of acupuncture can show us the entire effect of acupuncture.

### 2.1. Entire Effect of Acupuncture

Meridian is the “channel” which runs Qi and Xue (the theory of blood in TCM) and connects Zangfu (the viscera in TCM), body surface, and other parts of human body, regulating the body function. When a single acupoint in the “channel” is stimulated by acupuncture, an entire effect would be produced through the interactions during the transmit process of meridian Qi and pathopoeia factors [1]. As a global approach and a primary method of investigating biological phenotypes, omics could be utilized to explore the mechanism of acupuncture from the perspective of effect by revealing the overall alterations of molecular after stimulating on certain acupoints.

The omics researches on acupuncture are based on the researchers' understanding of diseases. On the basis of different disease knowledge and study purposes, various samples and omics methods are selected. Different omics researches reflect the integral cognition for study subjects from different aspects. Due to the variety of sample resources and omics methods, the conclusion of mechanism study on the same disease may differ. 

#### 2.1.1. Researches by Different Omics Approaches

Genomics, proteomics, and metabolomics had been applied in the study of acupuncture. The microarrays of either cDNA or oligonucleotide probes were used to screen for potential candidate genes to mediate acupuncture responses. The proteomic technologies of two-dimensional electrophoresis (2-DE) and mass spectrum (MS) analysis have been extensively used in acupuncture studies. Metabolic analysis has to deal with a highly diverse range of biomolecules which is different from genome and proteome analysis; recent advances in the two analytical platforms of mass spectrometry (MS) and nuclear magnetic resonance (NMR) spectroscopy have driven forward the discipline of metabolomics, but every platform covers only part of metabolomic [15]. In order to make metabolomic analysis to be a comprehensive research method as the genomic and proteomic assays, a community effort is required to develop the tools and databases and provide integration of these different tools and databases [16].

In a study reported by Gao et al. [17] the pain relieving effect of acupuncture may be mediated by endogenous opioid peptides in central nervous system. Sung et al. [18] analyzed the protein expression profile in hypothalamus by two-dimensional electrophoresis, which is different from the result of genomics research by Gao et al. Proteomics evidence indicated that the mechanism of analgesia by needling Zusanli was associated with inflammation, enzyme metabolism, and signal transduction. 

The researches indicated that experimental results based on high-flux omics studies may be false positive or false negative unavoidably, thus impacting further researches on acupuncture. In addition, it is still difficult to analyze the variance results of different samples and different omics methods and to systematically integrate these results. On the basis of omics studies, creating system models which could be driven by clinical data would help us to better understand the molecular mechanism integrally [19].

#### 2.1.2. Researches with Different Sample Sources

In the genomics study on the treatment effect of acupuncturing on GB34 (Yanglingquan) and LR3 (Taichong) with 1-methyl-4-phenyl-1,2,3,6-tetrahydropyridine (MPTP) induced Parkinson's disease animal models, Choi et al. analyzed the genetic changes in spinal cord [20] and Corpus Striatum [21] before and after acupuncture treatment based on gene chips technology and validate the results by reverse transcription-polymerase chain reaction (RT-PCR). It was proved that beneficial regulation on genes was developed by acupuncture, which showed their treatment effect by protecting nerves and inhibiting degradation of Corpus Striatum, respectively.

In a genomic study [22] on the pain relieving effect of electroacupuncture on Zusanli, the RNA changes in spinal nerves before and after acupuncture treatment were analyzed based on cDNA microarray technology. Signal transduction, gene expression, and an algesia pathway regulation were involved in the mechanism, which is different from the research by Gao et al. [17].

The effect of acupuncturing on specific acupoints is not only related to diseases but also to body conditions. In the genomics study [23] about acupuncture treatment on allergic coryza, gene expression profile in respect of positive/negative Phadiatop (Ph) test reaction [Ph(+) and Ph(−)] was analyzed before and after acupuncture treatment. Distinct physiological responses in Ph(+) and Ph(−) groups could be differentiated in the profiles. Another study [24] on the pain relieving effect of acupuncturing on the Hegu based on Genomics also indicated that individual differences of the pain relieving effect existed when specific acupoints were stimulated, and these differences were related to inheritance. The function of human body is mobilized fully to prevent and cure diseases by stimulating acupoints in the therapy of acupuncture, as the complexity of human body and disease determined the complexity of the acupuncture efficacy, analyzing the connections between effectors molecules and integrating that the omics results are the foundation of holistic understanding and evaluating acupuncture effect.

**Figure 1:**
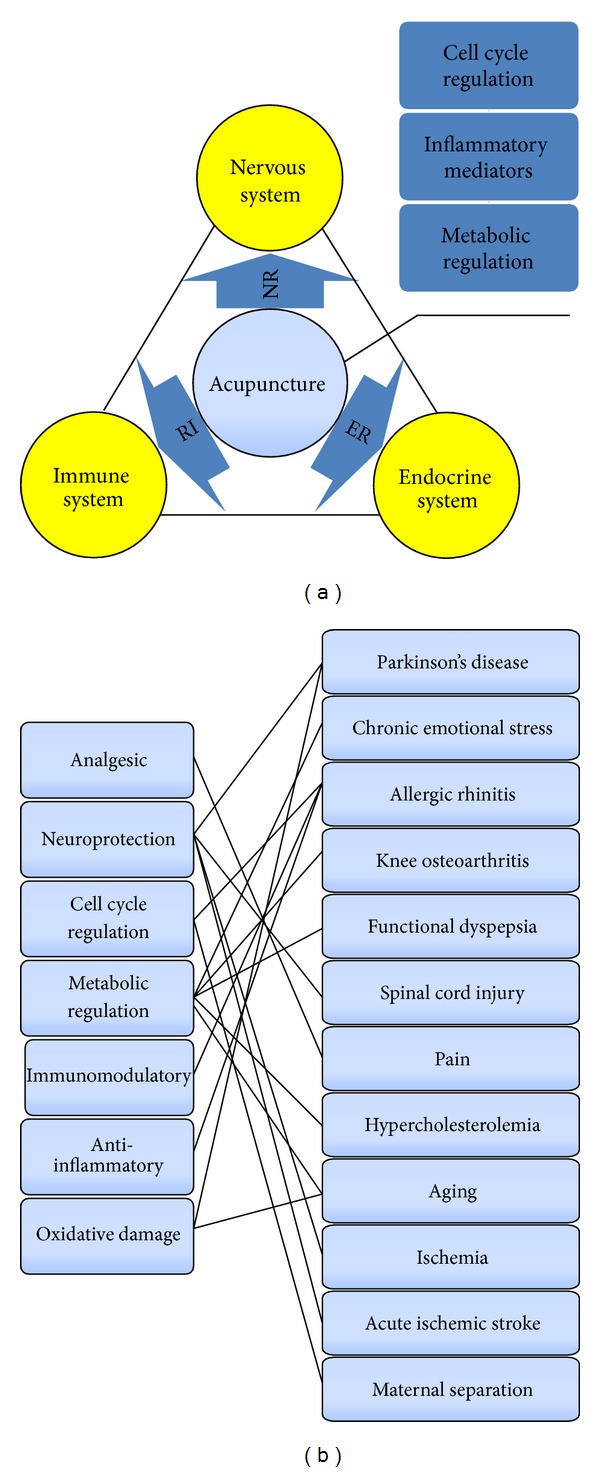
The entire effect of acupuncture is associated with the regulation of NEI which plays an extensive role in disease treatment. The nerve, endocrine, and immune systems are distributed over the body widely and the three systems can regulate mutually; thus the complex regulation network is formed and the other body systems are regulated. The body defense, growth, and development are regulated by the complex system (a). Acupuncture may treat diseases by regulating the NEI network and then develop effects such as anti-inflammation, neuroprotection, and antioxidative stress in disease treatment (b). Needling specific acupoints, the change of NEI network can reflect acupuncture effect systematically.

Omics studies on the acupuncture effects on Parkinson's disease [21, 22, 25, 26], rhinitis [24, 27], osteoarthritis [28], spinal injury [29, 30], pain [18, 19, 23, 25], aging [31, 32], ischemia (ischemic stroke) [33, 34], parturition [35] functional dyspepsia [36, 37], and so forth indicated that the mechanism of acupuncture is involved in the regulation of many body systems and is mainly associated with NEI system (Figure 1). The nerve, endocrine, and immune systems are distributed over the body widely. What is more, the three systems can regulate mutually through the common information molecules and receptors; thus the complex regulation network is formed and the other body systems are regulated. The body defense, growth, and development are regulated by the complex system (Figure 1(a)). Acupuncture may treat diseases by regulating the NEI network and then develop effects such as anti-inflammation, neuroprotection, and antioxidative stress (Figure 1(b)). Needling specific acupoints, the change of NEI network can reflect acupuncture effect systematically.

### 2.2. Special Effect of Meridian and Acupoint

Treatment by stimulating acupoints is the key point to distinguish acupuncture treatment from other therapies, and the special structure and function of the acupoints are the efficiency basis of acupuncture therapy. Wu et al. [37, 38] investigated the effects of acupuncture at Yangming meridian points and other meridian points using plasma and urine metabonomics approach based on ^1^H NMR and analyzed whether Yangming meridian points have common or different metabolic characteristics from other meridian points by pattern recognition. This study suggested that Yangming meridian points have different characteristics from those of both Yanglingquan and Weizhong. 

The treatment rules of acupuncture are very complicated but regular, which mainly depend on acupoints location, meridians attribution, and category [38]. The manifestations of acupoints specificity are diversified and compared with other specificities such as anatomy; efficiency specificity has more practical value and is more coincident with clinical requirements, which is regarded as the breakthrough point of acupoints specificity research. Omics researches show the global effect of acupuncture which possesses relative specificity; that is, needling different acupoints could treat the same disease and different diseases could be treated by needling the same acupoints.

#### 2.2.1. Same Acupoints for Different Diseases

Needling a specific acupoint could develop a widely biological effect; in the omics study of acupuncture, needling Zusanli could develop the effects such as immunity regulating [36, 39], pain relieving [18, 19, 23], and anti-inflammation [40], thus treating many diseases and developing more effects by synergy of other acupoints. While in TCM, the major effects by needling Zusanli are germinating Wei (the theory of stomach in TCM) Qi and drying Pi (the theory of spleen in TCM) dampness. 

Omics researches showed that acupuncture could treat diseases by regulating the NEI network, and the information molecules and their receptors shared by nervous, endocrine, and immune systems were associated with the occurrence and development of several diseases. Li et al. [14] found that heat and cold ZHENG were associated with the disorder of different NEI regulation models, which were able to respond to different TCM ZHENGs. Based on NEI network, they successfully establish heat and cold ZHENG model. Needling specific acupoints could treat several different diseases, from which the entire effect of acupuncture may be consistent with the regulation of certain TCM ZHENGs.

#### 2.2.2. Same Disease with Different Acupoints

**Figure 2:**
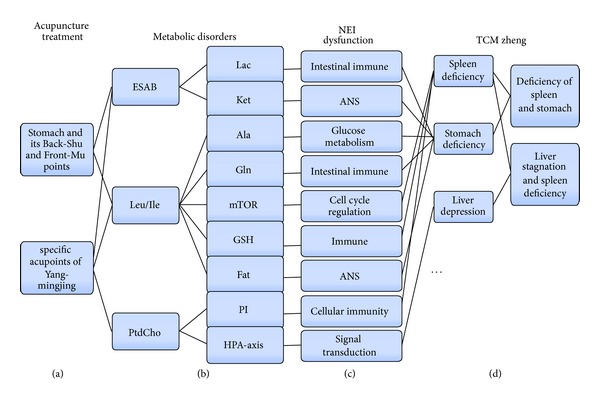
Special effect of meridian and acupoint. A plasma metabolomics investigation was designed to investigate the metabolic difference between FD patients and healthy volunteers, and a series of differential metabolites were sought out (b). The pathway analysis indicated that FD is related to some disorders in energy metabolism and especially to the NEI dysfunction (c) and the corresponding ZHENGs of FD are mainly regarded as Pi-Wei weakness or Gan depression and Pi deficiency (d). When the Wei and its Back-Shu and Front-Mu points as well as the specific acupoints of Yang-mingjing were needled, both of them have beneficial regulative effects on the metabolites associated with FD (a). The corresponding changes of NEI network reflect that different points selection methods may have certain regulative effects on different TCM ZHENGs, which might be the mechanism of Meridian Specificity.

Functional dyspepsia (FD) is a set of common symptoms including abdominal pain or discomfort. A plasma metabolomics research based on ^1^H NMR technology was designed to investigate the metabolic difference between FD patients and healthy volunteers, and a series of differential metabolites were sought out [41, 42] (Figure 2(b)). The pathway analysis indicated that FD was related to some disorders in energy metabolism and especially with the NEI dysfunction (Figure 2(c)). And the corresponding ZHENGs of FD were mainly regarded as Pi-Wei weakness or Gan (the theory of liver in TCM) depression and Pi deficiency [43] (Figure 2(d)). 

When the Wei and its Back-Shu and Front-Mu points [42] as well as the specific acupoints of Yang-mingjing were needled [43], both of them had beneficial and regulative effects on the metabolites associated with FD. And the regulative intensity was greater and the range was wider compared with that of the nonacupoints. Moreover, the longer the treatment time of acupuncture was, the more obvious positive regulative effects it would have. Therefore both methods had therapeutic effect on FD. As disease is complex and the practitioners may treat disease from different angles, they may select different acupoints when treating disease. There were differential regulative effects on potential biomarkers and key metabolites of FD when the stomach and its Back-Shu and Front-Mu points as well as the specific acupoints of Yang-mingjing were needled, so the regulative effects to FD-related NEI network were different; thus different point selection may make certain regulative effects on different TCM ZHENGs (Figure 2). The location of FD in TCM is in Pi and Wei, and the main therapeutic effects by needling the back shu points and the front mu points are in diseases of Zangfu. The ^1^H NMR metabolomics studies of FD with acupuncture treatment showed that needling the acupoints could regulate Qi, Xue, yin, and yang of the Zangfu, helping to restore normal physiological function of Zangfu. Acupuncture with selecting acupoints along meridians is the most important point selection method in the treatment of FD [44]. Meridians and collaterals are the channels of transporting the Qi and Xue, contacting Zangfu and body surface as well as all parts of the body, and it is the regulatory system of body function. When the specific acupoints of Yang-mingjing were needled, the treatment effect of selected acupoints along meridians was a whole regulation which was showed by ^1^H NMR metabolomics studies of FD. The stimulations acted on acupoints, and the acupoints were connected with meridians, so Yin and Yang, deficiency, and excess could be regulated through restoring Qi-Xue transportation.

Biology molecular alterations before and after acupuncture intervention could reflect the ZHENG information at certain structure and function level, which embodies different ZHENG classification of acupuncture. ZHENG is a complicated nonlinear system which emphasizes the interaction and relation of various factors, and it could be described as a model with dysfunctional relationship. Clinical ZHENG will present different interfaces by observing the disease appearance with different methods and points of view, which is called different ZHENG classification of TCM [45]. Needling specific acupoints is guided by meridian theories which could beneficially regulate the corresponding biology molecular of TCM ZHENG from the level of disease location or pathogenesis, which might be the mechanism of Meridian Specificity. 

The specificity rules of meridians and acupoints which are illustrated by researching are helpful to select acupoints for treatment in clinic and to make acupoints selection methods more in line with ZHENG classification. 

## 3. Omics Researches of Acupuncture Based on ZHENG Classification

**Figure 3:**
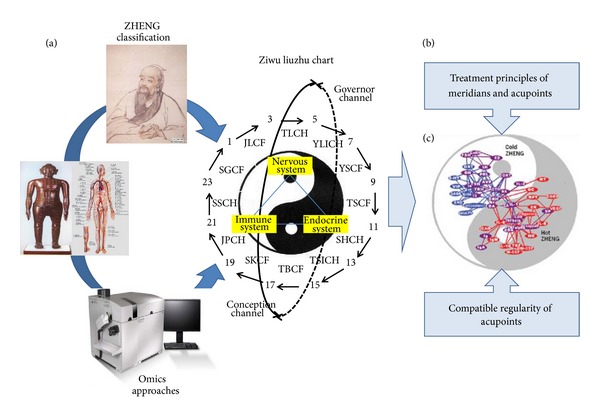
Understanding acupuncture with omics researches based on ZHENG classification. The omics researches of acupuncture based on ZHENG classification are the systematic research which are under the guidance of TCM theory and could bring the complexity of acupuncture effect and theory to light (a). By analyzing the connections among the ZHENG-related biological molecules in the omics studies of acupuncture, biology network models had been established for the further study of ZHENG. Studying the structure changes of biology network models would help us understand the action characteristics of acupoints and the molecular mechanism of their synergistic effect (b). (c) cited from [14].

ZHENG classification of acupuncture reflects the comprehensive and systematic understanding of disease. It is the theoretical basis of guiding acupoints selection, improving the clinical efficacy as well as studying the acupuncture. Omics researches of acupuncture based on clinical ZHENG classification could reveal the effect mechanism of acupuncture at molecular level integrally by ignoring the anatomic location of specific tissues and organs. By analyzing the connections among the ZHENG relevant biological molecules in the omics studies, biology network models had been established for the further study of ZHENG. Researching and analyzing the structure changes of biology network models, it would make objective evaluation for health status, ZHENG changes and treatment efficacy and it will help us understand the action characteristics of acupoints and the molecular mechanism of their synergistic effect (Figure 3).

### 3.1. Treatment Principles of Meridian and Acupoint

In the imparting and inheriting history of acupuncture, a theoretical system of ZHENG classification and treatment with its own characteristics which guide the diagnosis and treatment of acupuncture comprehensively and systematically was formed. Meridian theory is the key of the theory of acupuncture. It is the ZHENG classification according to meridian theories that clinical acupuncture ZHENG classification takes as the “main body,” ZHENG classification according to location as the “key,” ZHENG classification according to eight principles as the “guidance,” and ZHENG classification according to visceral theory as the “supplement” [46]. Omics researches based on different ZHENG classification can reflect the features and functions of acupoints in disease treatment.

Osteoarthritis (OA) belongs to “bone impediment” in TCM whose characteristics are deficiency in origin and excess in superficiality. According to the pathological sites of OA, by topical ZHENG classification, left and right Xiyan which is near knee joint were selected as treatment acupoints. Changes in gene expression of patients who were response to warm acupuncture before and after treatment were analyzed by gene chip technology; the result showed that genes related to inflammation were changed, which may be the direct treatment effect of needling left and right Xiyan in improving the relevant symptoms of OA [47].

Kidney-yang is the foundation of yang Qi. Kidney-yang deficiency is very common in clinic which mainly manifests as deficiency and cold of the whole body. According to the theory of TCM, the kidney masters the bones which has close relationship with the pathogenesis of OA. Previous studies also showed that kidney-yang deficiency was the highest incidence of TCM ZHENG pattern in OA [48]. With the analysis of etiology and pathogenesis of kidney-yang deficiency pattern by viscera ZHENG classification, the acupoints of Guanyuan and Qihai as well as warm needling method were selected to treat OA. Shen [49] thought that kidney-yang deficiency pattern was an overall performance of multisystems and organs dysfunction which are mainly related to the dysfunction of nervous system, endocrine system, and immune system. 

Ding et al. and Yang et al. [50, 51] found that signal transduction abnormalities among cells may play a key role in the development and progression of OA with kidney-yang deficiency. Various common signal molecules and receptors in nervous, endocrinology, and immune system are the molecule structure basis of NEI network. Genomics research about OA with deficiency cold by warm acupuncture treatment demonstrated that the genes of signal transduction were significantly expressed [47]. Taking Guanyuan, Qihai, and Zusanli as master points, traditional warm needling technique could stimulate Yang Qi of kidney and recover the physiological function of kidney by regulating NEI network so that the meridians are dredged and the symptoms of OA could be improved.

Quadriceps atrophy is caused by knee pain and joint dysfunction and vice versa. The Wei meridians being circulated through quadriceps, Yang-mingjing is rich in Qi and Xue; it nourishes the ancestral sinew [52]. Deficiency of Qi and Xue could lead to emptiness of meridians, which could be the reason of osteoarthritis. Based on the meridian differentiation, Zusanli was selected as the treatment acupoint. The modern research showed that Pi and Wei in TCM are closely related to immune theory in modern medicine [53]. The researches of genome [29, 53] showed that the pathogenesis of osteoarthritis are involved in abnormal expression of immune-related genes. Further studies conducted by Yang et al. [51, 54] showed that osteoarthritis belonging to Kidney-Yang deficiency is involved in 13 immune-related gene expression abnormalities. Needling Zusanli could induct or repress the expression of immune-related genes in order to restore the physiological function of Wei meridian of Foot-Yangming, which could develop the therapeutic effect on osteoarthritis by playing a regulative role in enriching the Qi, Xue, and meridians to make the therapeutic effect on OA.

By improving corresponding symptoms, different point selection methods have a direct or indirect therapeutic effect on disease treatment, taking a temporary solution and effecting a permanent cure.

### 3.2. Compatible Regularity of Acupoints

Different methods of ZHENG classification are overlapping at some extent in the clinical use of acupuncture while each method has its own merit and characteristic, so different ZHENG classification methods are irreplaceable in clinic [55]. By integrating data of omics researches guided by systems theory of ZHENG, it would help us understand the molecular mechanisms of acupoints compatibility. 

Genomics research showed that the mechanism of warm acupuncture treatment for OA with Kidney-Yang Deficiency may be related to the recovery of energy metabolism, inflammation, immune function, and signaling systems. Among them, the therapeutic effect of needling the left and right Xiyan and Yanglingquan may associate with the regulation of inflammation and immune-related genes. Needling Qihai and Guanyuan with the method of traditional warm acupuncture could beneficially regulate the metabolic changes of OA by affecting the NEI network. While the normal function of NEI network is dependent on the regular of synthesis, secretion and transport of the whole system, TCM theory holds that the transport and transformation of various substances in the body depend on the normal Pihealth movement. Thus, the warming acupuncture on Zusanli could enhance the overall regulative effect.

Acupuncture treatment is reasonably compatible according to the action laws of acupoints based on the comprehensive ZHENG classification. Acupuncture genomics studies showed that characterized acupoints which develop specific regulative effect on disease-related NEI network may form a new system through the interrelating and interacting of the network to treat diseases integrally.

## 4. Summary and Prospect

The effects of acupuncture are complex and it is unilateral to explore the mechanism of acupuncture in accordance with reductionism at the molecular levels due to the limitation of science. However, as the development of science, the essence of acupuncture may be uncovered at atom or electron levels [56]. At the molecular levels, omics researches of acupuncture based on ZHENG classification provided a wealth of information such as molecular structure information for the further systemic study of acupuncture.

Clinical ZHENG is a complex and nonlinear system which is consistent with the complicated and various properties of disease. While it is too complex for data processing which limits the further study of the ZHENG quantization and mechanism, and even through omics experiments, only small subsets of ZHENG correlated variables could be observed and most variables are still hidden which must be defined by computing [57], while the relative deficiency of data processing makes the omics data not easily be fully interpreted [58]. Therefore, in the omics researches of acupuncture based on ZHENG classification, we must decompose the ZHENG legitimately according to its property for further study [59].

With the application of bioinformatics and other science technologies, biological networks which connect and interact with each other should be established on the foundations of genomics, proteomics, and metabolomics in acupuncture study. With the researches on the relationship between different ZHENG classifications and the spatiotemporal distribution rule of nonspecific substances based on biological networks, the combination of omics technologies and different ZHENG classification will be truly achieved, and the molecular mechanism of the acupuncture will be understood in system levels. 

TCM can be regarded as traditional systems biology, and it is more important to discover the behavior of the system compared with the understanding of the structure of biological systems. System biology introduced the concept of perturbation as an artificial tool to control the state [60]. Under the artificial controlled state, dynamic characteristics of specific life system in different conditions and different times are studied. The integrated effect of acupuncture treatment is actually one of the manifestations of systems biology perturbation which is based on the endogenous substances, and as acupuncture is an important part of TCM, the omic study of acupuncture based on ZHENG classification has certain guidance and reference for the study and development of systems biology. 

## Abbreviations

Ala: Alanine
CNS: Central nervous system
ER: Endocrine regulation
ESAB: Energy supply and application bottleneck
Gln: Glutamine
GSH: Glutathione
HPA: Hypothalamic pituitary adrenal
Ile: Isoleucine
IR: Immune regulation
JLCF: Jueyin liver channel of foot
JPCH: Jueyin pericardium channel of hand
Ket: Ketone body
Lac: Lactate
Leu: Leucine
mTOR: Mammalian target of rapamycin
NR: Neuroregulation
Ph: Phadiatop
PI: Phosphatidylinositol
Pro: Proline
PtdCho: Phosphatidylcholine
SGCF: Shaoyang gallbladder channel of foot
SHCH: Shaoyin heart channel of hand
SKCF: Shaoyin kidney channel of foot
SSCH: Shaoyang Sanjiao channel of hand
TBCF: Taiyang bladder channel of foot
TLCH: Taiyin lung channel of hand
TSCF: Taiyin spleen channel of foot
TSICH: Taiyang small intestine channel of hand
YLICH: Yangming large intestine channel of hand
YSCF: Yangming stomach channel of foot.
